# Inhibition of *Helicobacter pylori* CagA-Induced Pathogenesis by Methylantcinate B from *Antrodia camphorata*

**DOI:** 10.1155/2013/682418

**Published:** 2013-01-08

**Authors:** Chun-Jung Lin, Yerra Koteswara Rao, Chiu-Lien Hung, Chun-Lung Feng, Hsien-Yuan Lane, David T. W. Tzeng, Ping-Ning Hsu, Chih-Ho Lai, Yew-Min Tzeng

**Affiliations:** ^1^Graduate Institute of Clinical Medical Science, China Medical University, Taichung 40402, Taiwan; ^2^Institute of Biochemical Sciences and Technology, Chaoyang University of Technology, Taichung 41349, Taiwan; ^3^Department of Biochemistry and Molecular Medicine, University of California Davis Comprehensive Cancer Center, Sacramento, CA 95817, USA; ^4^Department of Internal Medicine, China Medical University Hospital, Taichung 40402, Taiwan; ^5^Institute of Genomics and Bioinformatics, National Chung Hsing University, Taichung 40227, Taiwan; ^6^Graduate Institute of Immunology, College of Medicine, National Taiwan University, Taipei 10051, Taiwan; ^7^Department of Microbiology and Graduate Institute of Basic Medical Science, China Medical University, Taichung 40402, Taiwan

## Abstract

The bacterial pathogen *Helicobacter pylori* (Hp) is the leading risk factor for the development of gastric cancer. Hp virulence factor, cytotoxin-associated gene A (CagA) interacted with cholesterol-enriched microdomains and leads to induction of inflammation in gastric epithelial cells (AGS). In this study, we identified a triterpenoid methylantcinate B (MAB) from the medicinal mushroom *Antrodia camphorata* which inhibited the translocation and phosphorylation of CagA and caused a reduction in hummingbird phenotype in HP-infected AGS cells. Additionally, MAB suppressed the Hp-induced inflammatory response by attenuation of NF-*κ*B activation, translocation of p65 NF-*κ*B, and phosphorylation of I*κ*B-*α*, indicating that MAB modulates CagA-mediated signaling pathway. Additionally, MAB also suppressed the IL-8 luciferase activity and its secretion in HP-infected AGS cells. On the other hand, molecular structure simulations revealed that MAB interacts with CagA similarly to that of cholesterol. Moreover, binding of cholesterol to the immobilized CagA was inhibited by increased levels of MAB. Our results demonstrate that MAB is the first natural triterpenoid which competes with cholesterol bound to CagA leading to attenuation of Hp-induced pathogenesis of epithelial cells. Thus, this study indicates that MAB may have a scope to develop as a therapeutic candidate against Hp CagA-induced inflammation.

## 1. Introduction

 Chronic infection with the human bacterial pathogen *Helicobacter pylori *(Hp) causes gastritis and peptic ulceration and increases the carrier's risk of developing gastric cancer [1]. Hp possesses various virulence factors known to be important for the induction of disease during infection. One of the best described effectors molecule is cytotoxin-associated gene A (CagA) which translocated into host gastric epithelial cells by type IV secretion system (T4SS) [2, 3]. This T4SS represents a needle-like structure (also called T4SS pilus) protruding from the Hp surface and is induced by host cell contact to inject virulence factors including CagA [3]. CagA and the T4SS played a crucial role in gastric cancer development of gerbils [4]. Once intracellular, CagA localizes on the inner surface of the plasma membrane and becomes phosphorylated on tyrosine residues by Src family kinases [5]. The translocation and phosphorylation of CagA are triggered by direct interaction of the Hp T4SS with the *α*_5_ *β*_1_ integrin, which is found on the surfaces of epithelial cells [5, 6]. This interaction triggers the delivery of CagA into the host as well as subsequent Src kinase activation. The phosphorylated CagA (pCagA) subsequently induces a signaling cascade, resulting in the induction of proinflammatory responses in epithelial cells [5]. Previous reports described that pCagA leads to the activation of interleukin (IL)-8 transcriptions through the nuclear factor (NF)-*κ*B signaling pathway [3, 5–7]. Recently, CagA has been recognized as an oncoprotein that acts in mammals which were demonstrated by a transgenic mouse model [8].

Several reports demonstrated that translocation and phosphorylation of CagA are mediated by cholesterol-rich microdomains of the plasma membrane, commonly termed lipid rafts [9–11]. These domains not only are enriched in cholesterol but also contain phospholipids and glycosylphosphatidylinositol-anchored proteins and have been reported to be sites utilized by Hp to interact with host cells [12, 13]. It is known that Hp migrates toward and acquires exogenous cholesterol from the plasma membranes of host epithelial cells [9–11]. Cholesterol depletion can lead to reduction of CagA-induced pathogenesis in host cells [9]. Therefore, development of a new strategy to target bacterial virulence factor mediated by competing cholesterol-enriched binding sites was thought as a potential therapeutic approach.

 Current therapies for Hp infection are antibiotics based and often compromised by antimicrobial resistance. The search for new chemical compounds against Hp is warranted. Natural products have great potential to serve as sources of bioactive molecules and have been shown to influence the outcome of Hp infection [14]. Traditional medicines have been used to treat a wide range of ailments, including gastrointestinal disorders, such as dyspepsia, gastritis, and peptic ulcer disease [14]. *Antrodia camphorata* (AC), named “Niu-chang-chih” in Chinese, is a medicinal mushroom being widely used as a food dietary supplement for cancer prevention in several Asian and European countries [15]. Although, more than hundred secondary metabolites have been identified from Ac, particular attention has been directed to a triterpenoid, methylantcinate B (MAB). MAB has been reported to display tumor-specific cytotoxicity against various cancer cell lines [16]. Additionally, our recent study also indicates that MAB attenuates the Hp-associated inflammation in gastric epithelial cell line (AGS cells) [17]. However, the molecular mechanism underlying the inhibition of Hp-induced inflammatory responses is not yet completely dissolved.

 In this paper, we investigated the effects of MAB on CagA translocation and phosphorylation in Hp-infected AGS cells. In addition, we also examined the effects of MAB on CagA-induced inflammatory responses by the determination of NF-*κ*B activation, translocation of p65 NF-*κ*B, and phosphorylation of I*κ*B*α*, and IL-8 secretion. It is known that CagA interacts with membrane cholesterol leading to induction of inflammation in Hp-infected AGS cells. Since the chemical structure of MAB is similar to that of cholesterol, determining the binding mode of MAB to CagA should enhance our understanding of the pharmacophoric features of MAB responsible for its inhibitory activity against Hp CagA-induced inflammation. This led us to investigate the mechanism of MAB interaction with CagA by the competition of its binding to cholesterol.

## 2. Materials and Methods

### 2.1. Antibodies and Chemicals

Antibodies against CagA, ubiquitin, and *β*-actin were purchased from Santa Cruz Biotechnology (Santa Cruz, CA). For detection of CagA phosphorylation, 4G10 platinum antiphosphotyrosine was purchased from Millipore (Temecula, CA). Antiphospho-NF-*κ*B p65 (Ser536), anti-I*κ*B-*α*, PCNA antibodies were purchased from Cell Signaling Technology Inc. (Beverly, MA). MG132 was purchased from Sigma-Aldrich (St. Louis, MO). Lipofectamine 2000 and 4′,6-diamidino-2-phenylindole (DAPI) were purchased from Invitrogen (Carlsbad, CA). NF-*κ*B-Luc plasmid was purchased from Stratagene (La Jolla, CA), and the IL-8 promoter construct (IL-8/wild-type, nucleotides −162 to +44) was kindly provided by Dr. Chih-Hsin Tang of the Department of Pharmacology, China Medical University [18]. Luciferase substrate was purchased from Promega (Madison, WI). NE-PER nuclear and cytoplasmic extraction reagent kit was purchased from Thermo Scientific Inc. (Rockford, IL).

### 2.2. Cell Culture and Cytotoxicity Assay

Human gastric epithelial cell line, AGS cells, was cultured in Ham's F12 medium (Hyclone, Logan, UT) with 10% of decomplement fetal bovine serum (Hyclone, Logan, UT) and incubated in 5% CO_2_ at 37°C. All experiments related to bacterial infection were not supplemented with antimicrobial agents. The 3-[4,5-dimethylthiazol-2-yl]-2,5-diphenyl tetrazolium bromide (MTT) assay was employed to assess the cytotoxicity of MAB in AGS cells as described previously [17]. Treatment of AGS cells with maximum concentration of MAB (50 *μ*M) barely influenced the viability of AGS cells. 

### 2.3. *Helicobacter pylori* (Hp) Strains

Hp 26695 (ATCC 700392) wild-type strain containing CagA gene was used, and its isogenic mutant strain ΔCagA was generated as previously described [9]. Hp strain with CagA-EGFP was kindly provided by Dr. Ping-Ning Hsu of the Graduate Institute of Immunology, College of Medicine, National Taiwan University [19]. All Hp strains were cultured and maintained on Brucella agar plates (Becton Dickinson, Franklin Lakes, NJ) containing 10% sheep blood. Bacteria were cultured for 36–48 h and then added to epithelial cells at a multiplicity of infection (MOI) of 100.

### 2.4. Purification of Methylantcinate B (MAB)

The compound MAB used in this study was isolated from chloroform extracts of the fruiting bodies of *A. camphorata*, following the extraction and isolation procedures as we described previously [16, 17]. The purity of the MAB was >98% by HPLC. Its structure was confirmed by comparison of its mass and nuclear magnetic resonance spectral data with those in the literature [16, 17].

### 2.5. Western Blot Analysis

AGS cells (1 × 10^6^) were seeded in 6-well plates and performed assigned experiments. Cells were lysed with 100 *μ*L RIPA reagent (150 mM NaCl, 50 mM Tris Base pH 7.4, 1 mM EDTA, 1% NP-40, and 0.25% deoxycholate), and the supernatants were utilized to performed western blot assay. The relative of bands were quantified using ImageJ and UN-SCAN-IT software (Silk Scientific Corporation, Orem, UT) [20].

### 2.6. Hummingbird Phenotype Analysis

AGS cells (1 × 10^6^) were cultured in 12-well plates at 37°C for 24 h followed by infection with wild-type Hp strain 26695 at an MOI of 100 for 6 h. Elongated cells (hummingbird phenotype) were defined as thin needle-like protrusions that were longer than 20 *μ*m the detail of a typical elongated cell phenotype was described by pervious report [10]. All samples were performed in duplicate from three independent experiments. The proportion of elongated cells was calculated from the numbers of cells having the hummingbird phenotypes.

### 2.7. Luciferase Activity Assay

AGS cells (3 × 10^5^) were seeded in 12-well plates and transfect cells with either IL-8-Luc or NF-*κ*B-Luc plasmid by using Lipofectamine 2000. After 24 h incubation, cells were then cocultured with wild-type or ΔCagA strain of Hp for 6 h. To prepare total cell lysates, cells were washed once with PBS and 100 *μ*L of reporter lysis buffer (Promega) was added, and cells were scraped from dishes. Samples were centrifuge with 13,000 rpm for 5 min, and 80 *μ*L of luciferase substrate was added to 20 *μ*L samples. Luminescence was measured by using a 96-well microplate luminometer (ThermoLabsystems, Helsinki, Finland).

### 2.8. Analysis of IL-8 Secretion

AGS cells were grown to 90% confluence in 12-well plates and then experimental design was performed as described previously [21]. After 6 h incubation, cultured supernatants were collected, and the IL-8 concentration was determined by enzyme-linked immunosorbent assay (ELISA) by using a sandwich ELISA kit (R&D systems, Minneapolis, MN) according to the manufacturer's instructions.

### 2.9. Immunofluorescence and Confocal Microscopy Analysis

To visualize localization of Hp and the CagA-EGFP translocation, AGS cells (2 × 10^5^) were seeded on coverslips in six-well plates and incubated for 16 h. Cells were infected with Hp for 6 h and then washed three times with PBS and fixed with 3.7% paraformaldehyde (Sigma-Aldrich) for at least 1 h. The cells were then permeabilized with 0.1% Triton X-100 for 30 min and stained with DAPI (5 *μ*g/mL). The stained cells were then analyzed by confocal laser scanning microscope (LSM 510, Carl Zeiss, Göttingen, Germany) with a 100x objective (oil immersion, aperture 1.3). The quantification of fluorescence intensity was analyzed by Image J (US NIH) as described previously [18].

### 2.10. Structural Comparisons and Modeling

The model of CagA C-terminal was compared with all protein structures available in the DALI server (http://www.ebi.ac.uk/dali/). Structural comparisons with the cholesterol binding protein (Protein Data Bank code: 3IW1) [22], were carried out using the program Discovery Studio to superimpose C*α* atoms. Combined sequence and secondary structure alignments, the figure preparation was done with the program ESPript [23]. Structural figures were prepared with the program PyMol (http://www.pymol.org). To build the CagA C-terminal region with cholesterol and MAB model, the substrate cholesterol and MAB were docked using the program iGEMDOCK, version 2.1 [24]. First, the 3IW1 was chosen as the template for CagA C-terminal modeling. Residues from 645 to 1188 for CagA C-terminal region were modeled by Discovery Studio (http://accelrys.com/products/discovery-studio/). The binding site for docking was determined by considering the protein atoms located ≤10 Å from the binding residues of CagA C-terminal model. The default parameters of iGEMDOCK were used.

### 2.11. Dot Blot Analysis

Polyvinylidene fluoride (PVDF) membranes (Millipore, Billerica, MA) were prepared and immersed into PBS to eliminate methanol. A series concentrations of MAB (0, 10, 20, and 50 *μ*M) and cholesterol (0, 0.5, 1.0, and 2.0 mg/mL) were added onto membranes at the center of grid with vacuums. The membranes were blocked by 3% BSA in PBS for 1 h. RIPA-lysed-Hp (2 *μ*g/mL) were applied onto the membranes and incubated at 4°C for 16 h without shaking. The membranes were washed with PBS twice and incubated with goat-anti-CagA antibody (Santa Cruz Biotechnology) at 4°C for 16 h. The membranes were washed with PBS and then incubated with anti-goat-HRP antibody (Santa Cruz). After 1 h incubation, the membranes were washed, and the images were visualized by using Image Quant LAS-4000 (Fujifilm, Tokyo, Japan). The relative density of images was quantified by using UN-SCAN-IT software (Silk Scientific Corporation, Orem, UT).

### 2.12. Binding Inhibition Assay

Cholesterol and CagA binding inhibition by MAB was determined by dot blot analysis. RIPA-lysed-Hp (2 *μ*g/mL) were applied onto the PVDF membranes. DMSO, MAB (5 *μ*M), cholesterol (4 mg/mL), and various ratios of cholesterol : MAB (1 : 1–1 : 8) were added onto the membranes. The membranes were blocked in 3% BSA for 1 h, washed with PBS, and then incubated with 0.1 mg/mL recombinant rat Apo-A1 (Biovision, CA) at 4°C for 16 h. The membranes were incubated with rabbit anti-Apo-A1 antibody (Biovision), followed by probed with anti-rabbit-HRP antibody (Santa Cruz). Bound cholesterol was visualized by developing the blot and quantified as done for the dot blot.

### 2.13. Statistical Analysis

The Student's *t*-test was performed to calculate the statistical significance of experimental results between two groups. *P* < 0.05 was considered significant.

## 3. Results

### 3.1. MAB Attenuates Hp CagA Translocation and Phosphorylation

**Figure 1:**
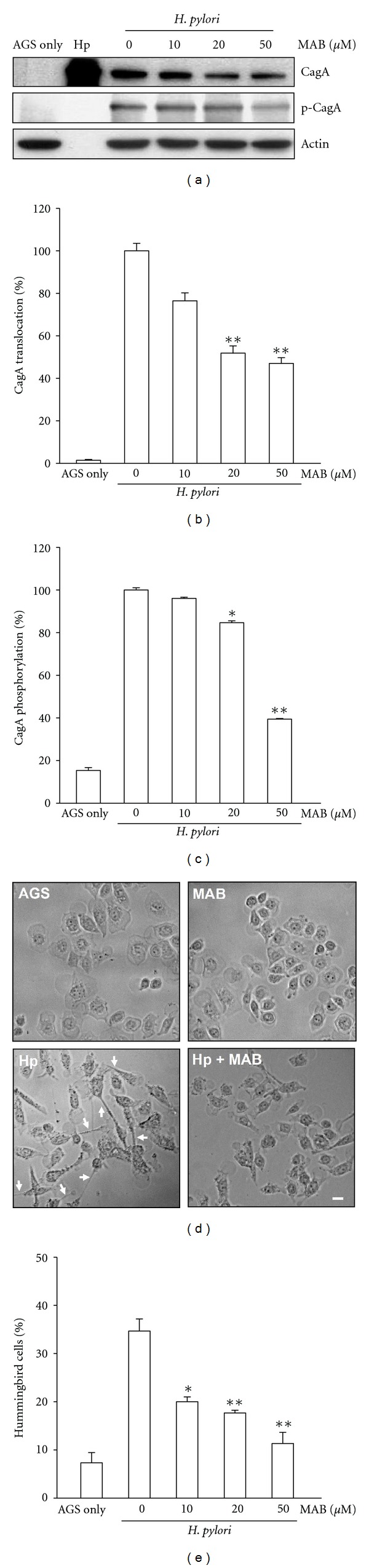
Methylantcinate B (MAB) influences *H. pylori* (Hp) CagA expression, translocation, and phosphorylation. AGS cells were treated with indicated concentrations of MAB prior infection by Hp at MOI 100 for 6 h. (a) The samples were immunoprecipitated for CagA and subjected to western blot analysis. Actin was represented as an internal control for equal loading. The levels of CagA translocation (b) and CagA phosphorylation (c) were determined by densitometric analysis. (d) The proportions of elongated cells were evaluated after cells cocultured with Hp and MAB for 6 h. (e) The hummingbird phenotype cells were determined. Results were shown as mean values ± standard deviations from 3 independent experiments. *P < 0.05 and **P < 0.01 were considered as statistically significant. Arrows in (d) represented elongated hummingbird phenotypes of AGS cells. Scale bar, 10 *μ*m.

CagA translocation is required in order to exert its effects on host AGS cells, and once within the host cytoplasm CagA can be phosphorylated by tyrosine kinases. Therefore, we first sought to determine if MAB treatment could affect the expression and function of Hp CagA phosphorylation and translocation in AGS cells. We utilized wild-type Hp (WT Hp) in this set of experiments. The levels of CagA translocation and tyrosine phosphorylation were analyzed by immunoprecipitation followed by western blot assays. MAB treatment for 6 h dose dependently (10, 20, and 50 *μ*M) reduced the levels of CagA protein expression compared to untreated cells (Figure 1(a)). Additionally, upon Hp infection the translocation and phosphorylation of CagA (pCagA) were increased to levels higher than those in noninfected cells (Figures 1(b) and 1(c)). However, with the MAB concentrations of 10, 20, and 50 *μ*M, the levels of translocated and phosphorylated CagA were dose dependently decreased (Figure 1(a)). In particular, when Hp-infected AGS cells was treated with 50 *μ*M of MAB, the levels of translocated CagA (Figure 1(b)) and phosphorylated CagA (Figure 1(c)) were reduced to 54.6% and 61.2%, respectively, as compared with nontreated cells. Next, we examined the effect of MAB on Hp-infected AGS cells polarity defect, known as the “hummingbird phenotype.” Induction of this characteristic loss of cell polarity (indicated by pronounced cell elongation) was clearly acquired in Hp-infected AGS cells (Figures 1(d) and 1(e)). In particular, in Hp-infected AGS cells, nearly 34.6% of AGS cells, exhibited the hummingbird phenotype as compared with the nontreated cells (Figures 1(d) and 1(e)). These data are consistent with the results obtained by western blot, suggesting that p-CagA molecule induces signaling that leads to profound AGS cell elongation. However, before treatment of Hp-infected AGS cells with MAB, the proportions of elongated cells were reduced in a dose dependent manner (Figure 1(e)). Specifically, the elongated Hp-infected AGS cells were reduced from 34.6% to 13.5% when pretreated with 50 *μ*M of MAB.

**Figure 2:**
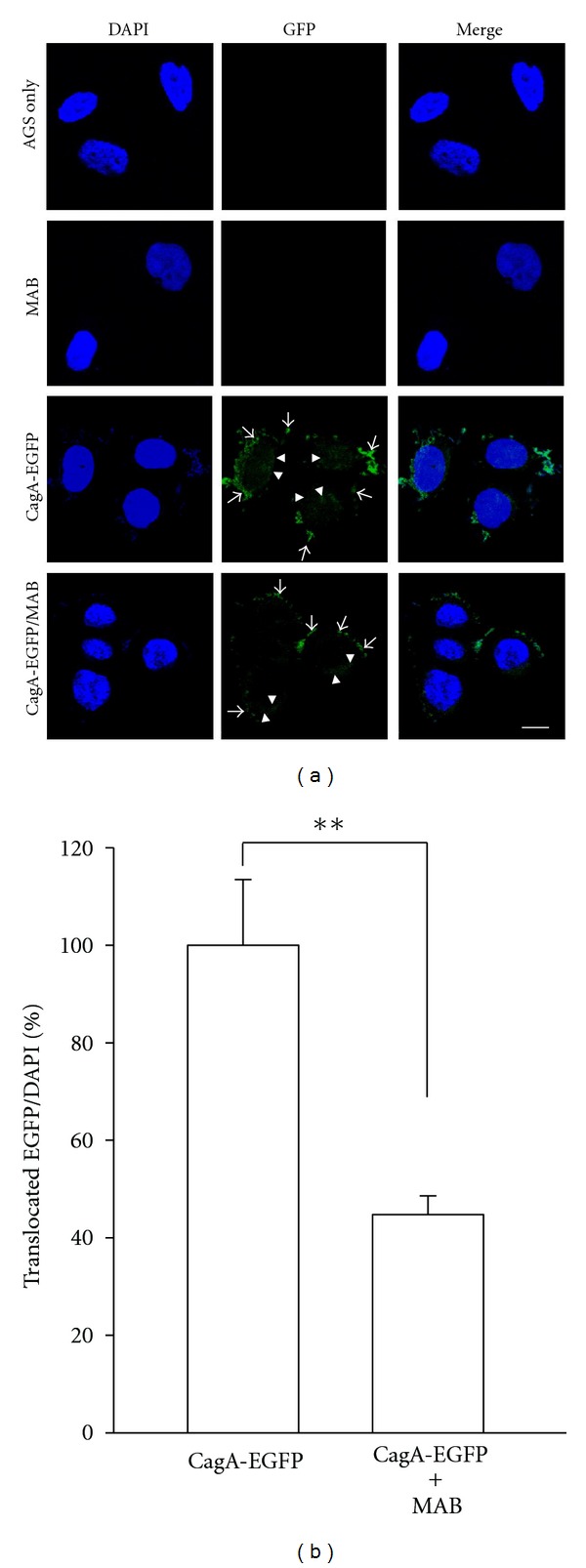
Translocation of CagA was attenuated by MAB. AGS cells were treated or untreated with MAB and infected with CagA-EGFP *H. pylori* at 37°C for 6 h. Cells were fixed and stained with DAPI (blue) to visualize bacteria and the cell nucleus. Samples were analyzed by confocal microscopy. The colocalization of GFP with DAPI appears cyan in the overlay to show bacteria (arrows). The translocated CagA-EGFP of green fluorescence signals (arrow heads) were quantified and normalized with DAPI signals. The data were analyzed using the Pearson correlation coefficient and presented in the right panel. Statistical significance was determined for 3 independent experiments. **P < 0.01; Bar, 10 *μ*m.

Given the above results, we then directly examined the inhibition of CagA translocation into cytoplasm by MAB using confocal microscopy. As shown in Figure 2, in cultured CagA-EGFP Hp with AGS cells, most of the Hp-associated CagA was delivered into the cells and localized in the cytoplasm. In contrast, when cells were treated with 50 *μ*M MAB, it was observed that major portion of CagA accumulated around the cells at sites of Hp infection. The distributions of fluorescence intensity for translocated CagA-EGFP signals in cytoplasm were determined and presented as intensity histograms (Figure 2, right panel). Our data clearly indicate that MAB has the activity to inhibit Hp CagA translocation in AGS cells.

### 3.2. MAB Inhibits Hp CagA Functions

**Figure 3:**
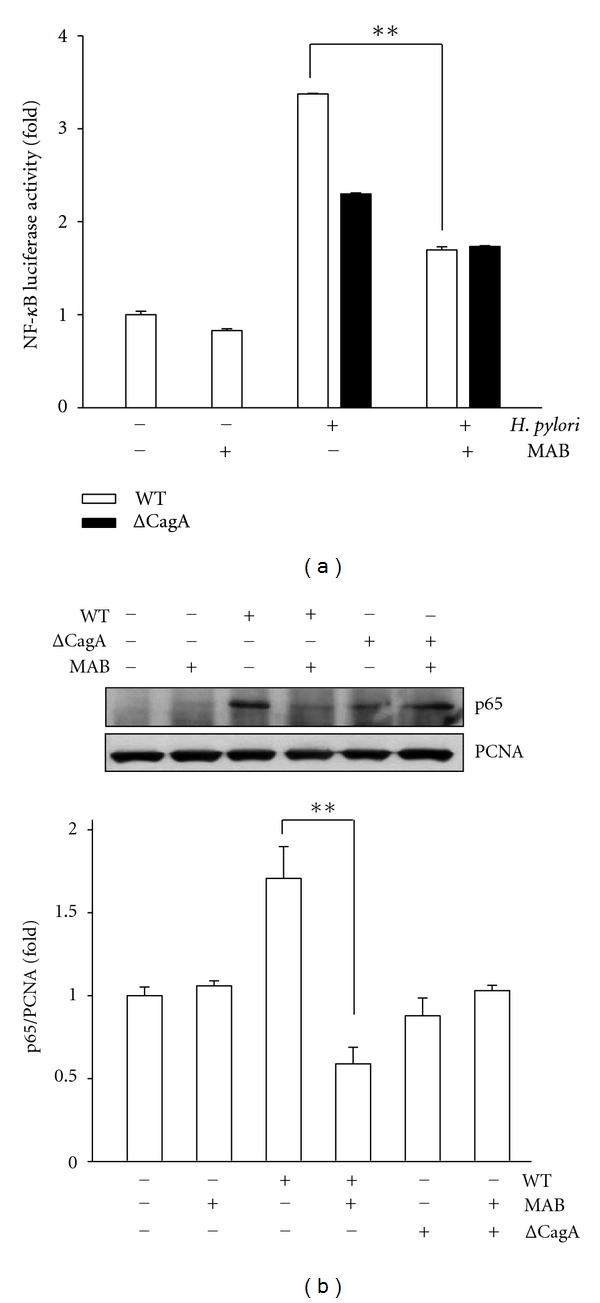
Effects of MAB on *H. pylori* (Hp) CagA-mediated NF-*κ*B activation and p65 nuclear translocation. The levels of NF-*κ*B luciferase activity (a) and nuclear p65 (b) were determined in the cell lysates as described in Section 2. Proliferating cell nuclear antigen (PCNA) was used as a loading control for the nuclear fraction of cell lysates. Results are shown as mean values ± standard deviations from 3 independent experiments. Statistical significance was determined using the Student's *t*-test when compared to the mock control. **P < 0.01 was considered as statistically significant. WT, wild-type Hp; ΔCagA,* cagA* mutant Hp.

Furthermore, we sought to identify the signal transduction pathway that might mediate CagA-dependent inflammation. Therefore, we next carried out transient transfection of AGS cells with NF-*κ*B-luc construct. The transfected AGS cells were treated with MAB and infected with WT Hp or the isogenic CagA-deleted mutant Hp (ΔCagA Hp). Treatment of AGS cells without WT Hp or with MAB alone, the expressions on the induction of NF-*κ*B activity, were at the basal levels (Figure 3(a)). Infection of AGS cells with WT Hp or ΔCagA Hp showed a noticeable increment in the NF-*κ*B luciferase activity. However, pretreatment of AGS cells with 50 *μ*M MAB reduced the levels of luciferase activity in samples exposed to WT Hp (Figure 3(a)). This phenomenon was minimal in ΔCagA Hp-induced luciferase activity as compared with WT Hp (Figure 3(a)). In line with this, we next examined the effect of MAB in p65 nuclear translocation, which plays an essential role in the regulation of NF-*κ*B activation [4]. We assessed the nuclear content of p65 NF-*κ*B in response to WT Hp or ΔCagA mutant Hp alone or preincubation with MAB. The results of immunoblots analysis revealed that incubation of the AGS cells with the WT Hp leads to an increase of p65 NF-*κ*B in the nuclear extracts as compared with mock treated or MAB alone or ΔCagA Hp treated cells (Figure 3(b)). However, in the presence of preincubation with 50 *μ*M MAB, the WT Hp-induced nuclear translocation of p65 NF-*κ*B was significantly diminished (Figure 3(b)). The quantitative data showed that 50 *μ*M MAB noticeably reduced the WT Hp-induced p65 translocation into the nucleus (Figure 3(b)). Together, these data indicate that MAB suppressed the Hp-induced translocation of p65 NF-*κ*B into the nucleus followed by attenuation of NF-*κ*B activation.

### 3.3. MAB Inhibits Hp CagA-Induced Phosphorylation of I*κ*B*α* and IL-8 Secretion

**Figure 4:**
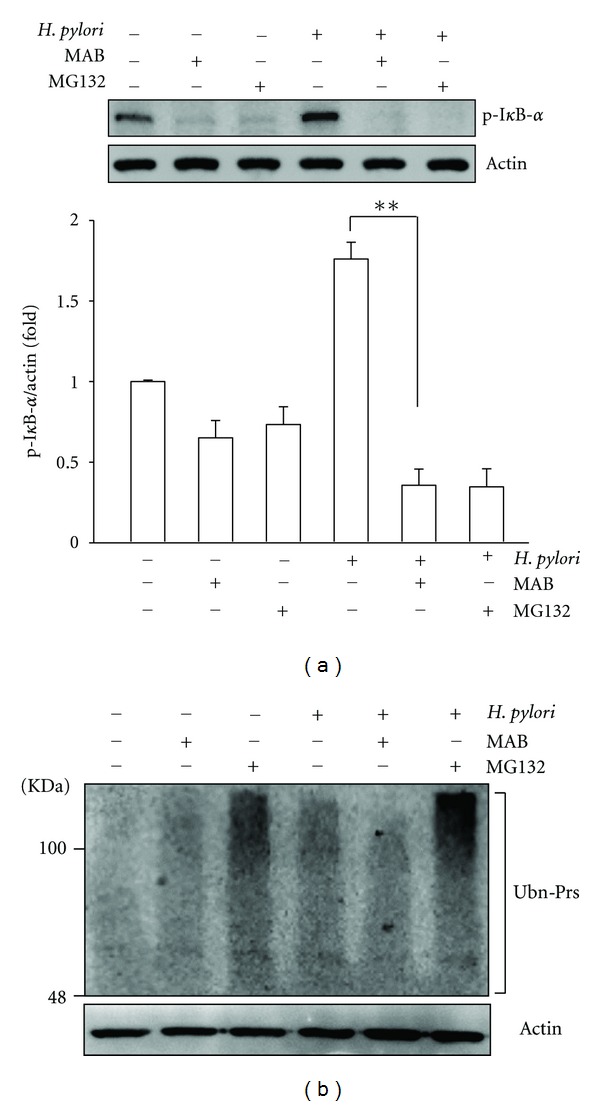
MAB prevents *H. pylori* (Hp) CagA-induced I*κ*B-*α* phosphorylation and protein ubiquitination. AGS cells were treated with MAB prior infection by Hp at MOI 100 for 24 h. (a) The cell lysates were subjected to western blot analysis. The levels of I*κ*B-*α* phosphorylation were determined by densitometric analysis. (b) The levels of protein ubiquitination were detected by western blot using a specific antibody to ubiquitin. Molecular mass markers (KDa) are shown on the left. Actin was represented as an internal control for equal loading. **P < 0.01 was considered as statistically significant. p-I*κ*B-*α*, phosphorylated I*κ*B-*α*; Ubn-Prs, ubiquitinated proteins.

**Figure 5:**
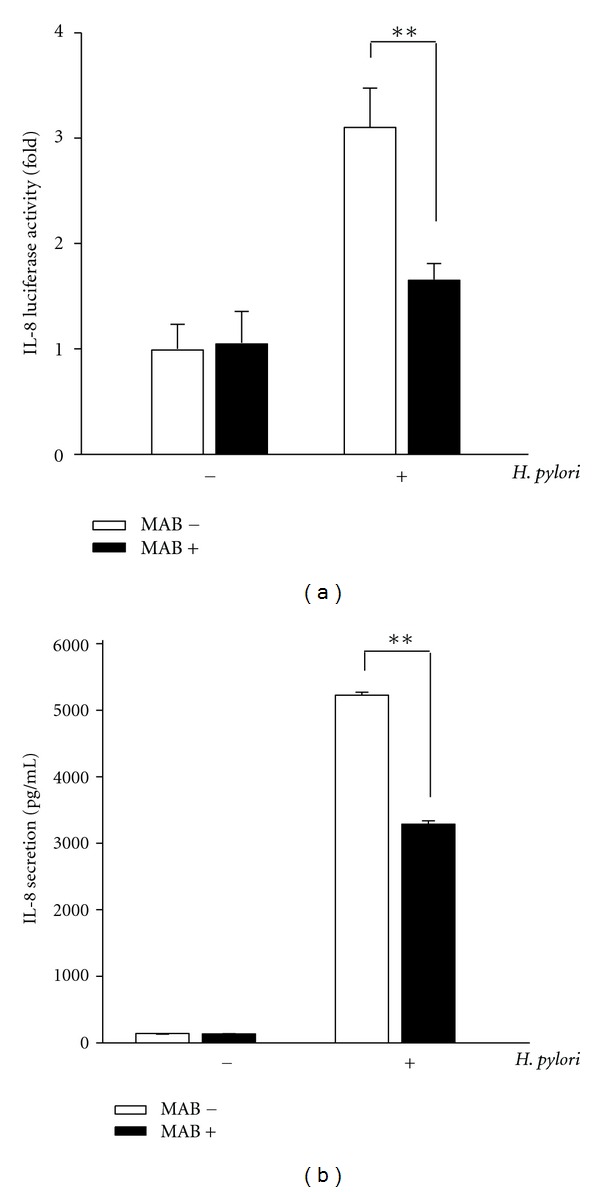
MAB attenuates *H. pylori*-induced IL-8 activity. AGS cells were infected with *H. pylori* in the absence or presence of MAB for 6 h. Cell lysates and culture supernatants were prepared for IL-8 luciferase activities (a) and IL-8 secretions (b), respectively. **P < 0.01 was considered as statistically significant. MAB−, not treated with MAB; MAB+, treated with MAB.

As NF-*κ*B activation requires phosphorylation and degradation of the inhibitory protein I*κ*B-*α* [5, 25], we exposed the AGS cells to WT Hp in the absence or presence of MAB, and the cell lysates were analyzed for I*κ*B*α* phosphorylation. Infection of AGS cells with WT Hp results in the increased level of proteasome target protein phosphorylation of I*κ*B*α*. As shown in Figure 4(a), the compound MAB and NF-*κ*B inhibitor (MG132) abolished the increased level of phosphorylated I*κ*B*α*. Additionally, MAB at 50 *μ*M also attenuated the accumulation of ubiquitinated proteins by Hp CagA in Hp-infected AGS cells (Figure 4(b)). Next, we examined MAB inhibitory effect on Hp-induced AGS cell IL-8 luciferase activity and secretion. As shown in Figure 5(a), parallel to the results obtained from NF-*κ*B measurements (Figure 3(a)), we observed a similar inhibitory effect on cytokine IL-8 luciferase activity. We also measured IL-8 protein levels in supernatants of AGS cells cocultured with WT Hp at an MOI of 100 for 6 h (Figure 5(b)). Hp-infected AGS cells grown without MAB produced 5316 pg/mL IL-8, a 11.4-fold increase over control cells. When Hp cultured in the presence of 50 *μ*M MAB were used, AGS cells generated only 3432 pg/mL IL-8, a 46.8% inhibition.

### 3.4. Structure-Based Virtual Docking of Cholesterol and MAB for CagA Model

**Figure 6:**
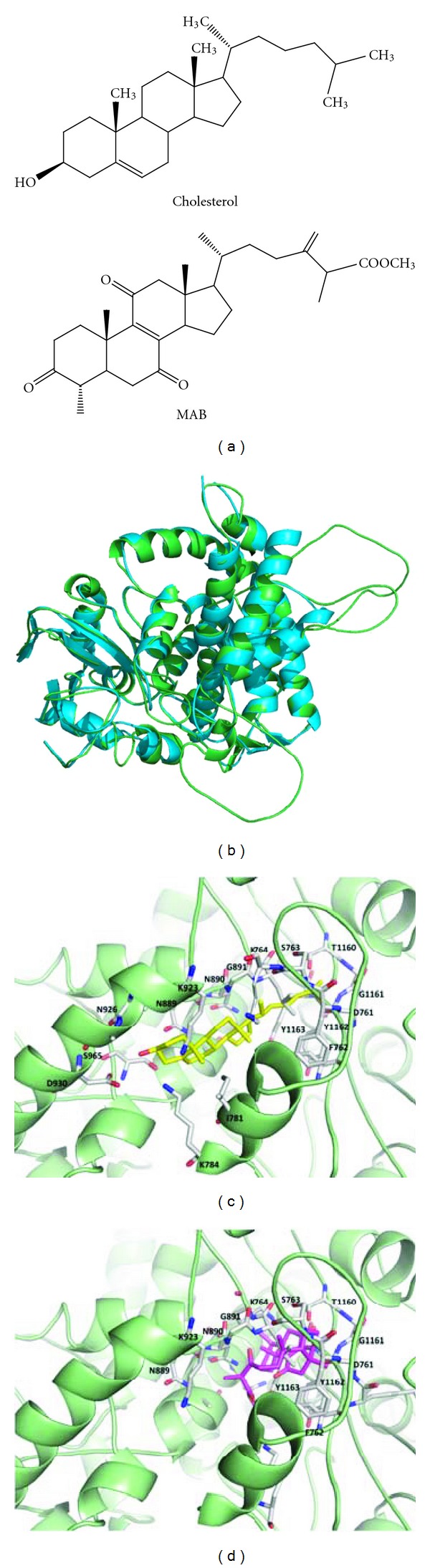
Structural comparisons and modeling simulations for cholesterol and MAB binding to CagA. (a) Chemical structures of cholesterol and triterpenoid MAB. (b) The overall structure of CagA C-terminal domain colored in green and cholesterol binding protein (PDB code: 3IW1) colored in cyan. (c) The model of CagA C-terminal region with cholesterol (stick, colored in yellow). (d) The model of CagA C-terminal domain with MAB (stick, colored in magenta). The numbers of amino acids shown in CagA were directly contacted with the cholesterol (c) or MAB (d) binding sites.

Once Hp's intimate contact with AGS cells is established, the T4SS of Hp further interacts with specific host cell surface molecules including cholesterol in lipid rafts to facilitate injection of CagA translocation and proinflammatory signaling. As the chemical structure of MAB resembles with cholesterol (Figure 6(a)), we hypothesized that MAB may bind with CagA C-terminal domain similar to that of cholesterol. This led us to investigate molecular modeling simulations to examine the interaction of cholesterol and MAB with CagA C-terminal domain. Structural comparisons with CagA and the cholesterol binding protein (Protein Data Bank code number: 3IW1) were then chosen as the template for CagA C-terminal region modeling (Figure 6(b)). By using iGEMDOCK, the structural modeling showed that cholesterol binding site of CagA model took the shape of a hydrophobic pocket (Figure 6(c)). Accordingly, as shown in Figure 6(d), a MAB molecule was inserted into the CagA C-terminal domain. Comparing their contact residues between the interaction of cholesterol and MAB with CagA C-terminal domain, the data showed 12 amino acids in the same interaction sites of CagA. The putative binding residues shown in CagA C-terminal region were D761, F762, S763, K764, N889, N890, G891, K932, T1160, G1161, Y1162, and Y1163. 

### 3.5. Interaction of CagA with Cholesterol Was Inhibited by MAB

**Figure 7:**
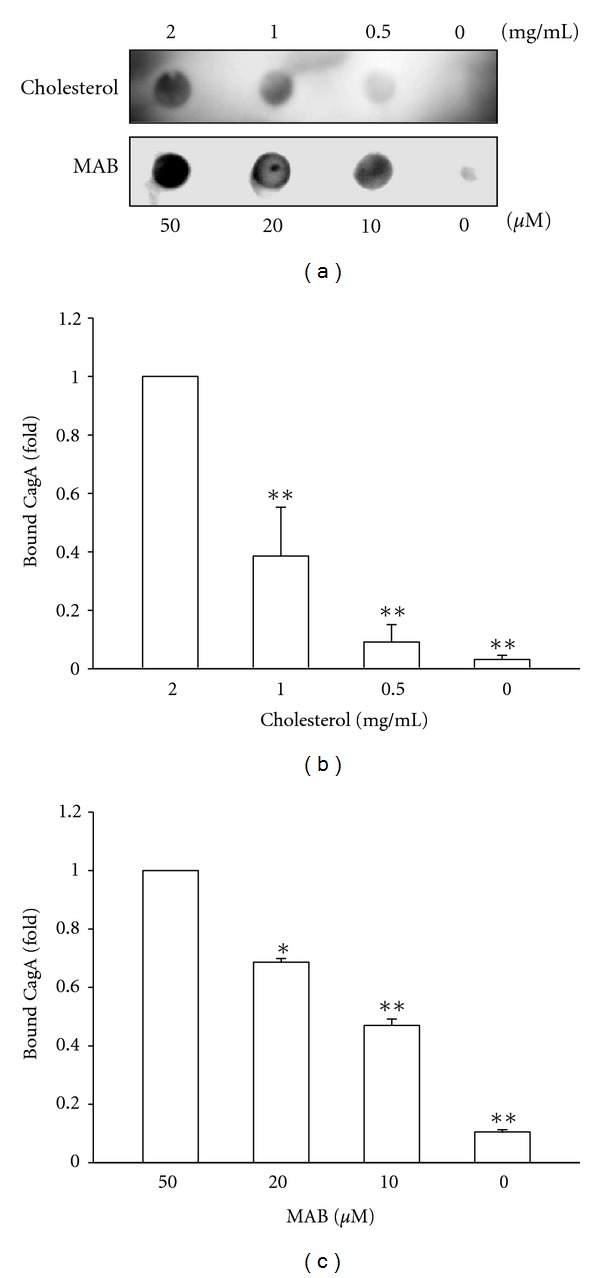
Binding of CagA to the immobilized cholesterol and MAB. (a) Representative dot blot that shows binding of CagA to various concentrations of cholesterol and MAB. The binding activities of CagA to the immobilized cholesterol (b) and MAB (c) were quantified by densitometric analysis with 3 independent experiments. *P < 0.05; **P < 0.01 compared to each highest concentration group, as determined by Student's *t*-test. Chol, cholesterol.

The structure-based simulations raised the possibility that CagA directly binds to MAB which may compete the interaction of cholesterol with CagA upon Hp infection. To explore this idea, series concentrations of cholesterol and MAB were prepared and overlaid onto PVDF membranes. As shown in Figure 7, CagA binds to various concentrations of immobilized cholesterol (0.5–2 mg/mL). Similarly, binding of CagA to the immobilized MAB was also shown in a dose-dependent manner (10–50 *μ*M). The results from the dot blot analysis indicate that both of cholesterol and MAB are able to bind CagA, consistent with the observation in structural simulations.

**Figure 8:**
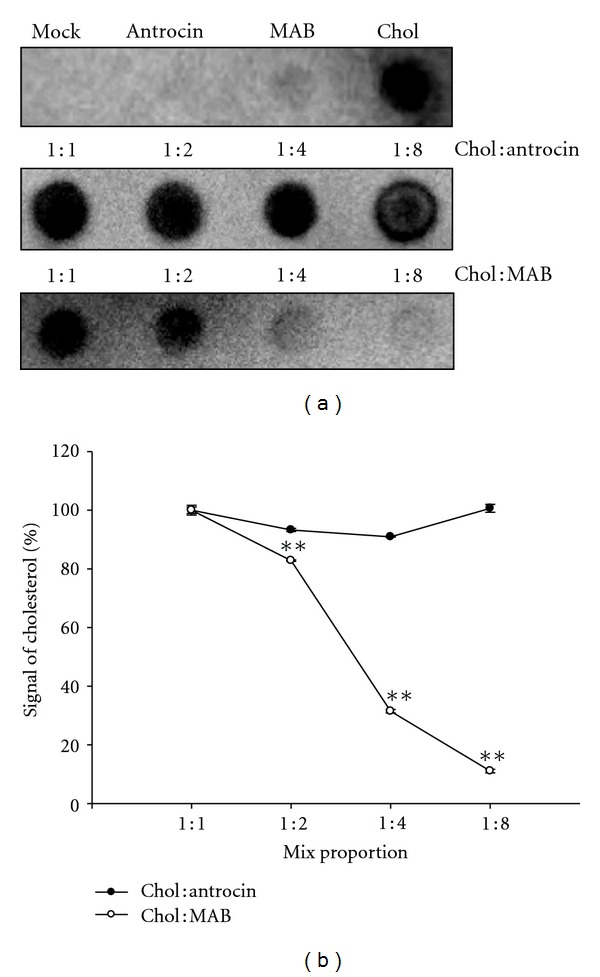
Binding of cholesterol to CagA was inhibited with increased levels of MAB. (a) A series of dot blots with various ratios of cholesterol : antrocin and cholesterol : MAB (1 : 1 to 1 : 8) were overlaid to the immobilized CagA and detected by antibody as described in Section 2. (b) The binding inhibition analyses were quantified and the results were representative as 3 independent experiments. **P < 0.01 was considered as statistically significant. Chol, cholesterol.

To further investigate the competitive effect of cholesterol and MAB, the binding inhibition assay was performed. CagA was dotted onto PVDF membranes, and a series ratio of cholesterol and MAB were added (cholesterol : MAB, 1 : 1, 1 : 2, 1 : 4, and 1 : 8). As shown in Figure 8(a), as MAB proportion raised, the amount of cholesterol bound to CagA was decreased as expected. The cholesterol signal was quantified, and the competitive effect dropped significantly from 82.9% to 18.5% (Figure 8(b)). To explore whether MAB has an exclusive inhibitory effect on cholesterol binding to CagA, a series ratio of cholesterol, and antrocin, a sesquiterpene lactone from Ac were added, and the binding inhibition assay was performed. It was found that antrocin does not affect the cholesterol binding to CagA (Figures 8(a) and 8(b)). These results reveal that MAB may interfere with the interaction between cholesterol and CagA by competitive with the binding site of CagA.

## 4. Discussion

Although Hp is not an intracellular pathogen, its interactions with host cells may provide certain survival benefits for Hp and contribute to the establishment of a chronic infection. Indeed, Hp uses host plasma membranes as a site for replication and formation of microcolonies [4]. Additionally, colonization of the epithelial cell membranes by Hp is enhanced when the apoptotic response of AGS cells is impaired. Epidemiological studies reveal that Hp physically interacts with AGS cells and introduces CagA protein into the host cells [4]. CagA is known for its role as inducer of proinflammatory responses in AGS cells, and as such, it enhances the activation of IL-8 through NF-*κ*B signaling pathway [3, 5]. The triterpenoid compound MAB has recently reported for its anti-inflammatory activities in Hp-infected AGS cells [17]. However, there is no study addressing the detailed protective mechanism of MAB in Hp CagA-induced pathogenesis. For the first time, we now provide the mechanism responsible for MAB inhibited Hp-induced inflammation by directly targeting CagA and this feature is likely by competing the cholesterol interaction with CagA and, thereby, attenuates CagA function in AGS cells.

 Current literature indicates that CagA molecules are directly translocated into AGS cells via a Hp T4SS [3, 8]. It is also reported that CagA is rapidly tyrosine phosphorylated after its injection into host AGS cells and mimics a host cell factor for the activation or inactivation of some specific intracellular signaling pathways [3]. Glu-Pro-Ile-Tyr-Ala (EPIYA) motifs in the C-terminal region of CagA protein have been identified as phosphorylation sites using site-directed mutagenesis of injected and/or transfected CagA [3]. Increased CagA signal intensity could result from increased amounts of injected CagA molecules undergoing phosphorylation at a specific site and/or by increased phosphorylation of multiple sites per CagA molecule. Accordingly, in this study we found that the compound MAB dose-dependently reduced the CagA translocation and phosphorylation (Figures 1(a)–1(c)). The most striking Hp-induced morphological change to host AGS cells is the induction of an elongated cell phenotype termed hummingbird [1]. This phenotype results from two successive events: the induction of cell scattering and cell elongation. The induction of early cell motility mainly depends on a CagA-dependent T4SS factor and cell elongation is clearly triggered by phosphorylated CagA. This phenotype may impact pathogenesis by influencing immune responses, wound healing, metastasis, or invasive growth of cancer cells* in vivo* [26, 27]. In this study, MAB dose-dependently suppressed the number of elongated cells (Figure 1(d)). Taken together, this data indicate that exposure to MAB affects the CagA expression, translocation, and leading to a reduction in the amount of CagA phosphorylation followed by reducing the hummingbird phenotype of AGS cells.

 Next, a hallmark of Hp infection is increased chronic inflammation. Previous reports have been demonstrated that CagA is an important determinant for the induction of transcription factor NF-*κ*B and this induced NF-*κ*B has a dominant role in IL-8 production in AGS cells [5]. The induced NF-*κ*B can be activated by phosphorylation via different signaling pathways leading to subsequent proteolytic degradation of I*κ*B [3]. Additionally, activated NF-*κ*B translocates to the nucleus where it upregulates IL-8 gene transcription [4, 5]. Therefore, the inhibition of NF-*κ*B signaling is an ideal strategy for eradication of Hp infection. In this study, MAB suppressed the NF-*κ*B activation, translocation of p65 NF-*κ*B into the nucleus, and phosphorylation of I*κ*B-*α* (Figures 2–4). Thus, these results indicate that MAB attenuated Hp CagA-induced inflammation through the NF-*κ*B-mediated signaling pathway. On the other hand, they are consistent with previous report that constitutive activation of NF-*κ*B was thought to be associated with progression of several tumor-cell types [1]. Here, we showed that MAB not only suppresses Hp CagA-induced NF-*κ*B activation but also blocks p65 nuclear translocation (Figure 3), implicating that MAB harbors anticancer activity. These results may provide molecular evidence for our previous report that MAB displayed cytotoxicity against various cancer cell types [16]. On the other hand, it is known that the expression of IL-8 in response to Hp infection is a key cause of Hp-induced inflammation and IL-8 is regulated by NF-*κ*B [5, 25]. In this stydy, MAB suppressed the IL-8 luciferase activity as well as its secretion (Figure 5), indicating that the immunostimulatory effect of the Hp was lessened by exposure to MAB. Collectively, these data indicate that pretreatment of AGS cells with MAB inhibited the Hp CagA-induced inflammatory response.

Previously it is reported that the natural triterpenoids possess potent serum cholesterol level reduced activities [15, 28, 29], indicating that triterpenoids have the potential in antihyperlipidaemia through modulation of cholesterol synthesis. Additionally, a previous study demonstrated that Ac can downregulate 3-hydroxy-3-methylglutaryl-CoA (HMG-CoA) reductase, which is a key enzyme for cholesterol synthesis [30]. However, in this study, our preliminary data showed that MAB which was isolated from Ac did not alter the level of cellular cholesterol (data not shown). This evidence indicated that MAB-attenuated CagA function was not through the depletion of cholesterol in membrane rafts.

 Several studies have been found that CagA C-terminal domain containing EPIYA motif in response to CagA targeted membrane rafts [10, 31]. Our recent report showed that tethering of CagA to cholesterol-enriched membrane microdomains is important for IL-8 induction in AGS cells [31]. Modulation of cellular cholesterol levels alter the partitioning of CagA into membrane rafts [9, 11, 13], indicating that CagA may interact with membrane cholesterol. Analysis of amino acid sequence of CagA revealed that some cholesterol-recognition/interaction-amino-acid-consensus-pattern- (CRAC-) like regions were represented in C-terminal domain (GenBank accession number: AE000511.1). The CRAC region contains the conserved motif L/V(X)1-5Y(X)1-5R/K which may contribute to the association of protein with cholesterol [32]. We then carried out the docking simulations using iGEMDOCK (version 2.1) [24]. Our results indicated that the cholesterol and MAB similarly interacted with CagA (Figure 6). Moreover, the same contact regions of 12 amino acids were localized in the C-terminal domain of CagA (Figures 6(c) and 6(d)). These results support our hypothesis that the direct binding of MAB to CagA lead to preventing cholesterol from interaction with CagA.

 In conclusion, we have shown that a triterpenoid MAB attenuated the Hp-induced inflammatory responses by competing the binding of cholesterol with CagA. We also demonstrated that CagA-induced pathogenesis was inhibited by treatment of cells with MAB. Thus, MAB may have a scope to develop as potential candidate against Hp CagA-mediated pathogenesis. However, further studies are necessary to investigate the importance of these findings *in vivo*.
